# Foliar application of esculin and digitoxin improve the yield quality of salt-stressed flax by improving the antioxidant defense system

**DOI:** 10.1186/s12870-024-05626-z

**Published:** 2024-10-15

**Authors:** Hemmat I. Khattab, Mervat Sh. Sadak, Mona G. Dawood, Fatma M. A. Elkady, Nesma M. Helal

**Affiliations:** 1 Botany Department, Faculty of Science, Ain Shams University, P.O.11566, Abbassyia, Cairo Egypt; 2 Botany Department, Agricultural and Biological Research Institute, National Research Centre, P. O. 12622, 33 El-Buhouth Street, Dokki, Giza Egypt

**Keywords:** Salinity, Flax, Digitoxin, Esculin, Defense mechanism, Yield components

## Abstract

**Background:**

Secondary metabolites of several plants, including esculin and digitoxin, which are cardiac glycosides, were previously employed for their therapeutic effects. The current study aims to investigate the functions of the main Na^+^ /K^+^ transport inhibitor digitoxin and the antioxidant esculin for enhancing flax plant growth and production under salinity.

**Methodology:**

Flax plants were irrigated with distilled water supplemented with 0.0 and 5000 mg/L salt solution starting from 15 DAS from sowing. Then exogenous treatment with digitoxin and esculin with 50 mg L^− 1^ and 100 mg L^− 1^ were used for this work.

**Results:**

According to the results of this work, foliar spraying of esculin or digitoxin increased the salinity tolerance of flax plants.The foliar application of either esculin or digitoxin induced an elevation in the contents of photosynthetic pigments, osmolytes including soluble sugar and proline as well as the total phenols in salt-stressed flax plants. Moreover, esculin and digitoxin in particular counteract oxidative stress by increasing the activity of antioxidant enzymes including superoxide dismutase, catalase, peroxidase, phenylalanine ammonia-lyase, and tyrosine ammonia lyase, leading to a decrease in reactive oxygen species and lipid peroxidation levels and electrolyte leakage. The efficiency of esculin and digitoxin to sustain ion homeostasis by inhibiting Na^+^ absorption and increasing potassium, calcium, and phosphorus in flax plants may be the reason for their protective actions towards salinity.As a consequence, esculin and digitoxin increased yield quantity and quality as shown by increases in all investigated yield criteriaas shoot height, root length, their fresh and dry weights as well asseed yield/plant (g), and 1000 seeds weight, especially those that improved the desired oil properties.

**Conclusion:**

In conclusion, this study concluded that digitoxin was more effective in inhibiting Na^+^ build-up and increasing flax salinity tolerance, particularly at the high investigated dose as compared to esculin. In this study, we reported the recent findings of exogenousapplication of either digitoxin or esculin glycosides which are new investigated salt alleviators never used before for improving the salt tolerance in flax plants.

## Background

Salinity is among major environmental stressors, that has a detrimental effect on plant growth, yield quantity and quality [1]. Excessive watering, decreased precipitation, increased surface evaporation, ion exchange, and insufficient agricultural methods have all contributed to a notable rise in soil salinity [2 & 3]. By 2050, it was predicted that *greater* than half the world´s arable land will be salinized [4]. Salinity negatively affects plant growth via osmotic stress stimulation and ion toxicity thereby stimulating production of reactive oxygen species (ROS) which provokes oxidative damage of nucleic acids, proteins, and lipids and causes the degradation of photosyntheticpigments as well as peroxidation of cell membranes [5 & 6]. Indeed, salinity altered the physiological and metabolic processes of plants as specified by reductions in water uptake, chlorophyll content, photosynthesis, transpiration rate, nutrient availability, stomatal conductance, and root hydraulic conductance [7]. Consequently, plants evolved a number of defense strategies to withstand salinity and sustain growth under stressful conditions. The antioxidant defense system includes antioxidants (ascorbic acid, glutathione, carotenoids andphenols) and antioxidant enzymes such as catalase (CAT; EC 1.11.1.6.), superoxide dismutase (SOD; EC 1.15.1.1) peroxidase (POD; EC 1.11.1.7) to buffer the accumulated ROS brought by salinity [8 & 9]. High levels of antioxidants as well as antioxidant enzymes in fact confer plant tolerance and lessen oxidative stress injuries [8–10].

Additionally, stressed plants accumulate compatible solutes, including carbohydrates, free amino acids, and proline to sustain the ionic balance of vacuole, remove free radicals and thereby protect macromolecules and plant organelles [11 & 12]. Proline can serve as a compatible solute, osmoprotectant, source of nitrogen and carbon, membrane stabilizer, and ROS buffering scavenger [11 & 13]. Furthermore, ion homeostasis (especially Na^+^/K^+^) is crucial for the survival of salt-stressed plants. It was hypothesized that the most significant factor affecting plant´s ability to tolerate salt, and as a result, the improvement of crops is the uptake, efflux, translocation, and compartmentation of harmful ions particularly, Na^+^ and Cl^−^ [13].

Furthermore, plants tolerate stressed conditions by promoting the synthesis of a variety of secondary metabolites [15 & 16]. Secondary metabolites function as signals or regulatory molecules, solutes, antioxidants, or ROS scavengers, then in order to help plants to tolerate stress [16]. Salinity has an impact on the metabolic pathways that lead to the accumulation of secondary metabolites such phenols, saponins, flavonoids, carotenoids, lignin, cardiac glycosides, etc [17].

Cardiac glycosides, in particular, are secondary metabolites that were utilized earlier to treat heart diseases [18, 19]. Digitoxin **i**s a cardiac glycoside utilized to treat and control congestive cardiac insufficiency, arrhythmias, and heart failure. As a consequence of digitoxin´s inhibition of the Na-K-ATPase membrane pump, intracellular sodium and calcium levels rise. The activation of contractile proteins may be facilitated by elevated intercellular calcium levels Moreover, sodium and potassium ions are actively transported both within and outside of cells by the enzyme Na^+^/K^+^-ATPase sodium ions outside cells [20]. It was discovered that the primary physiological role of digoxin is to establish and maintain the electrochemical gradient across the plasma membrane, which is necessary for biochemical processes like brain communication, osmotic control of cells, and ion homeostasis [23]. Numerous organic and inorganic compounds engage in secondary translocation as a result of this gradient potential [24 & 25].

Aesculin, or esculin, is a coumarin glucoside that naturally occurs in chestnut trees. Esculin is used as a vasoprotective agent (Drugs.Com.). Esculin is also used in microbiology laboratory to aid in the identification of bacterial species [23]. Esculin plays an important role in capillary protection, as it improves capillary permeability and instability. Indeed, esculin also showed good antioxidant properties, protecting triglycerides against auto-oxidation at high temperatures and used as an anti-inflammatory as well as making it suitable for after-sun treatments (https://go.drugbank.com/drugs/DB13155).

Flax is one of the oldest useful plants that have been cultivated for fiber and oil. Flax (*Linum usitatissimum* (Linn.), belongs to the family Linaceae. Notably, it has been recorded that flax is recognized as an excellent source of micronutrients, dietary fiber, protein, vitamin B1, lignan, and essential fatty acids (EFA), namely linoleic and α-linolenic acids [24]. Moreover, it has been reported that, the salt stress negatively affected seed yield and photosynthetic pigments includingchlorophylls a, b and carotenoid contents in flax plant [24].

The exogenous application of digitoxin and esculin glycosides is a novel strategy for improving and enhancing salinity stress tolerance in flax plants. Nonetheless, no research has been conducted about digitoxin and esculin glycosides applying to flax under salinity stress conditions, and many points need to be elucidated about the effects of digitoxin and esculin glycosides on flax salinity stress. Since flax plant as mentioned before is a multifunctional plant because all the plant parts have economic important so this study aimed to increase the cultivated flax area and thereby plant yield particularly flax seed and fibre. The objectives of this study were to clarify whether flax application of digitoxin and esculin glycosides can alleviates alt stress and induce stimulation of flax growth, from the perspective of morphology, physiology, and biochemistry. These results are expected to provide new insight into the physiological and biochemical mechanisms of digitoxin and esculin glycosides as a growth promotor and stress toleranceinducer and serve as fundamental data for agriculture applications.

Thus, the present investigation aims to examine the best and most efficient alleviators of salt stress, the major Na^+^ /K^+^ transport inhibitor digitoxin or the antioxidant esculin as well as their protective mechanisms against salt stress.

## Materials and methods

### Experimental procedure

In the wintertime of 2021/2022 a pot experiment was conducted in the National Research Centre’s greenhouse, Dokki, Cairo, Egypt, to explore the impact of Esculin and Digitoxin foliar spraying on flax plants exposed to saline water irrigation. Across the study, daytime temperatures varied between 12.3 and 17.0 °C, during the research with a mean of 15.4 ± 1.0 °C. The minimum and maximum temperatures were 17.8 and during the day 28.4^o^C and were 8.7 and 16.7^o^Cduring nighttime. The mean daytime temperature was 23.1 ± 2.8 °C, with a mean of 49.7% ± 9.2%. Daytime relative humidity ranged from 22.1 to 59.2%. Flax var. Letwania-9 with similar size and color were selected and sterilized for nearly 2 min with 1% sodium hypochlorite and then rinsed in running water. Ten identical air-dried seeds were planted in plastic pots containing seven kg of clay soil homogenized with sand with a 3:1 ratio (v/v). The physical and chemical properties of soil were determined. The soil had a clay-loam texture, with coarse sand at 1.4%, fine sand at 31.7%, silt at 39.6%, and clay at 27.3%, electrical conductivity (EC) 1.82 dS m-1, pH 7.5, organic matter at 1.93%, CaCO_3_ 7.88%, and available N, P, and K accounting for 45.6, 7.8 and 415.0 mg Kg^− 1^, respectively. The soil was fertilized three days prior to planting with the following: (1) ammonium sulfate (20.5% N) at an 800 Kg ha-1 dose; (2) super phosphate (15% P_2_O_5_) at a 240 Kg/hectare dose; and (3) potassium sulfate (48% K_2_O) at a 120 Kg/hectare dose which was thoroughly incorporated into each pot. Soil water holding capacity, 0.36, was determined by saturating the soil in each pot with water, letting it drain for 48 h, and weighing it. The water holding capacity was maintained at about 90%. Following the guidelines provided by the Egyptian Ministry of Agriculture and Land Reclamation all guidelines’ practices related to flax production were followed out. The experiment was factorial in a completely randomized design and replicated three times. The experiment comprised two factors. The first factor included two saline water levels namely 0.0, and 5000 mgL^− 1^ (according to a preliminary germination experiment using different concentrations of Esculin and Digitoxin (0, 25, 50, 100, 150 mg/L) and series of salinity levels namely, 1000, 2000, 3000, 4000, 5000, 6000 and 7000 mgL^− 1^ and choose 5000 mgL^− 1^ were conducted. Then appropriate concentrations of Esculin and Digitoxin (0, 50, 100 mg/L) under 5000 mg/L salinity level based on the results of growth characters). The preparation of salt mixture was done according to Stroganov’s [25] equation as in Tables (1 & 2). The pots were irrigated with equal volumes of various salinity levels.

**Table 1:** The constituents of the salt mixture used for chloride salinization expressed as % of the total salt content(g)

MgSO_4_	CaSO_4_	NaCl	MgCl_2_	CaCO_3_
10	1	78	2	9

**Table 2:** The constituents of specific anions and cations in the chloride mixture expressed as a percentage of total milli equivalents (from the molecular weights of the used salts)

Na^+^	Mg^+ 2^	Ca^+ 2^	SO^− 2^	Cl^−^	CO^− 2^
38	6	6	5	40	5

The second factor involved foliar application of different concentrations of Esculin and Digitoxin (0, 50, 100 mgL^− 1^) were applied at 30 and 45 days. We used 5000 mgL^− 1^ according to the results of a preliminary experiment on flax plants using different levels of salt irrigation water and treated with different concentrations of foliar treatment of Esculin and Digitoxin, according to the results of this experiment, we evaluate the salt stress tolerance of the flax variety used. Pots were divided into two groups, each one irrigated with one of the following salt concentrations (0.0 or 5000 mgL^− 1^), each pot received one liter of each used salt water. Therefore, the experiment consisted of ten treatments as combinations of two salt irrigation and five foliar (control, 50, 100 mgL^− 1^ of Esculin and 50, 100 mgL^− 1^ of Digitoxin) treatments. After plant emergence, flax seedlings were thinned ten days after sowing (DAS), and four plants per pot were left. Pots were irrigated with distilled water supplemented with 0.0 and 5000 mgL^− 1^ salt solution starting from 15 DAS from sowing of flax plants. Spraying flax plants with different concentrations of Esculin and Digitoxin (0, 50, 100 mgL^− 1^) was done at 42 and 56 days. Plants were sprayed from both sides of the row to achieve adequate coverage.

### Measurements

Plant samples were collected after 75 days from sowing, to assess the morphological parameters including shoot length (cm), fresh and dry weight (g/ plant), root length (cm), and root fresh weight (g) as well as some biochemical analyses. Flax plants were pulled when signs of full maturity appeared, then left on the ground to attain complete drying. The capsules were removed carefully. At harvest, plant height (cm), fruiting zone length (cm), technical stem length (cm), number of fruiting branches/plant, number of capsules/plants, biological yield/plant (g), seed yield/plant (g), and 1000 seeds weight (g), were recorded on random samples of 4 plants. Some biochemical analyses of the yielded seeds were done such as oil%, carbohydrates%, and protein.

### Biochemical analysis

Photosynthetic pigments: One gram of fresh leaves was homogenate with 85% (v/v) acetone, then the homogenate was filtered and made up to total volume (100 mL) by acetone. The absorbance was documented at three wavelengths of 663, 644, and 452.5 nm. Chlorophylls a & b and carotenoids were measured [26] as mg g^− 1^ fresh weight (FW). Total soluble sugars (TSS) were extracted and determined by the method described by Chow and Landhausser [27]. Extraction of TSS was performed with boiled 80% (v/v) ethanol. Then, the extract was filtered and evaporated, followed by dissolving the remaining residue in a known volume of distilled water to get ready for soluble sugar estimation which it has been achieved by anthrone reagent, and finally, the absorbance was measured at 620 nm. Furthermore, the method used for the total free amino acids were determined according to the method described by Sorrequieta [28]. So, the extract (0.1 ml) was mixed with 1.6 ml of ethanol/acetone (1:1 v/v), 0.1 ml phosphate buffer (0.5 M, pH 6.5) and 2 ml ninhydrin were pipetted into quick fit glass tubes. The tubes were placed in a boiling water bath for 20 min, then cooled immediately in ice water and methanol was added up to 10 ml. The absorbance was measured directly at 580 nm using spectrophotometer.Proline content was determined by Kalsoom [29]. Two mL of extract was added to 2 mL of acid ninhydrin reagent and 2 mL of glacial acetic acid. The absorbance was recorded against toluene as blank at 520 nm. Phenolic contents were determined and expressed as mg tannic acid g^_1^ FW as described by Gonzalez [30]. The extraction method of total phenols was achieved by 80% methanol, after evaporation, the evaporated residue was made up to the known total volume by distilled water. The estimation method was performed by Folin-Cicalteu reagent. The absorbance was recorded at 725 nm. Indole acetic acid (IAA) content was determined by the method of Larsen [31]. The fresh tissue was homogenate in cold 85% ethanol, and an electric stirrer was used to complete the extraction with 85% ethanol at approximately 0 °C. The solvent was changed three times. After filtration, the combined extracts were concentrated under a vacuum at 20–25 ◦C to a few mills. Then, IAA acid was determined by using gas liquid chromatography (GLC) in GVC pyeUnicam gas-liquid chromatograph equipped with dual flame ionization detector and dual channel recorder.

Membrane stability index MSI (%) was measured according to the method recorded by Karimi [32]. Electrolyte leakage (EL%) was determined according to Vahala [33].Leaf samples (0.5 g) were immersed in 20 mL of deionized water for about 24 h at 25 ◦C. Then, the electrical conductivity (EC) was measured for solution (L1) by using a conductivity meter. Samples were then re-immersed in deionized water at 120 ◦C for 20 min and then the final EC (L2) was measured after equilibration at 25 ◦C. Finally, EL was calculated from the following equation: EL% = (L1/L2) × 100.

The level of lipid peroxidation was measured by determining the malondialdehyde (MDA) content using the method of Hodges [34]. The reaction of MDA was performed by thiobarbituric reagent. The absorbance was measured at two wavelengths; 532 and 600 nm.

Hydrogen peroxide level was determined calorimetrically according to Yu [35]. The H_2_O_2_ concentration was expressed as µmol g^− 1^FW. Superoxide anion radicals (O^−•^_2_) were determined according to the method described by Doke [36].

### Assay of enzymes activities

Enzyme extractions were collected following the method described by Chen and Wang (2006). Peroxidase (POX) (EC 1.11.1.7) activity was determined by Kumar and Khan [37]. Superoxide dismutase (SOD) (EC 1.12.1.1) activity was assayed at 560 nm by nitro-blue-tetrazolium (NBT) reduction method using spectrophotometer (VEB Carl Zeiss) [38]. Catalase (CAT) (EC 1.11.1.6) activity was determined spectrophotometrically by following the decrease in absorbance at 240 nm [38]. The enzyme activities were calculated by Kong [39]. Lipoxygenase (EC 1.13.11.12) activity was estimated according to Doderer [40] and expressed as unit(1 nmol of substrate oxidized per minute) per mg enzyme protein. Determination of PAL and TAL Activity were estimated by Haard and Wasserman [41].

Minerals content of Na^+^, P, K^+^, Ca^2+^, Mg^2+^ were determined according to the method described by Chapman and Pratt [42].

### Seed chemical analysis

The oil of seeds was extracted by using Soxhlet apparatus as described by Das [43]. Total carbohydrates were estimated according to Albalasmeh [44]. The total protein concentration was determined according to the method described by Bradford [45]. Analysis of fatty acids was determined from seed oil specimenswhich collected from the second seasonseeds [46]by using gas liquid chromatography of methyl ester using a “HEWLETT PACKARD HP 6890 series GC system” instrument equipped with a flame ionization detector (FID). The capillary column “HP-INNOWAX polyethylene glycol”; length (30 m); diameter (530 mm) and film thickness (1 μm). Two injections were made from each sample. The operating conditions were: initial temp. 120ºC; final temp. 240ºC and detector temp. 300ºC. The nitrogen, hydrogen and air flow rates were 30, 30 and 300 ml/min respectively as described by Fedak and De La Roche [47].

### Fiber analysis

At full maturity, flax-defoliated plants were collected for the retting process as described by El - Hariri [48]. Then the separated fibers were combed with a special comb as reported by El - Hariri [48]. The fiber quality as evident in terms of fiber length (cm), fiber yield/plant (g), fiber% and fiber diameter in µ was determined. Cellulose% was determined according to the method of Updegraff [49]. The fiber lignin% was determined by the method described by Collings [50].

### Statistical analysis

Analyses of variance (ANOVA) for all data presented in this investigation were calculated using SPSS v20.0 (SPSS Inc., Chicago, USA) analyzing software. Statistical significances of the means were compared with Duncan’s test at *p* ≤ 0.05 levels [51], the standard error (SE) of the means presented in tables, and the figures are means ± SE (number of replicates = 3).

## Results

### Plant growth

The growth of flax plants was significantly affected by salinity (Table 3). The height, and fresh weights, of shoots as well as shoot dry weights of salt-stressed flax plants, were significantly reduced as compared with their corresponding unstressed plants In contrast salinity stimulated root length and root fresh weight as compared to unstressed control plants (Table 3). Foliar application of either digitoxin or esculin at concentrations of 50 mgL^− 1^ and 100 mgL^− 1^ significantly enhanced the measured growth parameters in control unstressed plants as well as in salt-stressed plants as being compared with their corresponding controls (unstressed and salt-stressed untreated control). Such effects were positively related to glycoside concentrations. The greatest increases in growth parameters were measured in flax plants treated with 100 mg/L digitoxin in the absence and presence of salinity as compared with the other treatments.

**Table 3:** Impact of different concentrations of either esculin or digitoxin (50, 100 mgL^− 1^) on some growth parameters of flax plants in the absence or presence of salinity stress (0.0, 5000 mgL^− 1^). Each value is the mean of ten replicates ± SE with P value < 0.05. The values with the same letter in the same column are non-significant and the values with different letters are significant

Salinity (mg/L)	Treatment	Conc.	Shoot length (cm)	Shoot fresh wt (g)	Shoot dry wt (g)	Root length (cm)	Root fresh wt (g)
		mgL^-1^					
0. 0	Control	0	37.66 ± 0.08^fg^	1.626 ± 0.02^d^	0.404 ± 0.006^g^	11.00 ± 0.57^hij^	0.369 ± 0.008^h^
	Esculin	50	44.66 ± 1.45^d^	1.836 ± 0.03^c^	0.627 ± 0.003^d^	13.66 ± 0.66^efg^	0.424 ± 0.004^g^
		100	54.33 ± 0.66^b^	2.146 ± 0.01^b^	0.761 ± 0.02^b^	14.33 ± 0.66^def^	0.513 ± 0.006^e^
	Digitoxin	50	49.33 ± 0.33^e^	2.112 ± 0.08^b^	0.689 ± 0.008^c^	14.66 ± 0.33^cde^	0.521 ± 0.002^de^
		100	56.66 ± 0.33^a^	2.502 ± 0.02^a^	0.811 ± 0.004^a^	16.00 ± 0.57^bc^	0.637 ± 0.003^c^
5000	Control	0	28.33 ± 0.66^ij^	0.900 ± 0.01^g^	0.274 ± 0.005^k^	13.66 ± 0.33^efg^	0.458 ± 0.01^f^
	Esculin	50	35.66 ± 0.88^gh^	1.203 ± 0.03^f^	0.395 ± 0.01^gh^	15.66 ± 0.33^bcd^	0.542 ± 0.01^d^
		100	42.00 ± 1.00^e^	1.407 ± 0.009^e^	0.518 ± 0.003^f^	18.33 ± 0.33^a^	0.695 ± 0.006^b^
	Digitoxin	50	38.66 ± 0.33^f^	1.229 ± 0.03^f^	0.421 ± 0.003^g^	16.66 ± 0.33^b^	0.635 ± 0.006^c^
		100	45.66 ± 0.33^d^	1.560 ± 0.03^d^	0.594 ± 0.009^e^	18.66 ± 0.33^a^	0.837 ± 0.01^a^

Results are means of three replicates. ± show SE

### Photosynthetic pigments

Salt stress variously affected the photosynthetic pigment levels in flax plants (Table 4). The imposition of salt stress at 5000 mg/Lcausedreductions in chlorophyll a& b as well as the total pigment content of the flax leaves compared to those of the unstressed plants (Table 4). However, the carotenoid levels were significantly increased under salt stress. The foliar application of either digitoxins or esculin markedly increased chlorophylls a, b, and carotenoid levels under the two salt levels. The increments in pigment levels in digitoxin and esculin-treated plants were concentration-dependent. The maximum values of chlorophyll a&b, total pigments, and carotenoid levels were recorded in high-dose digitoxin-treated plants (100 mgL^− 1^) as compared to the othertreatments.

**Table 4:** Impact of different concentrations of either esculin or digitoxin (50, 100 mgL^− 1^) on photosynthetic pigments, chlorophyll a (Chl a), chlorophyll b (chl b) and carotenoids (car) µg/ g fresh wt. of flax plants in the absence and presence of salinity stress (0.0, 5000 mgL^− 1^). Each value is the mean of three replicates ± SE with P value < 0.05.The values with the same letter in the same column are non-significant and the values with different letters are significant

Salinity mgL^− 1^	Treatment	Conc. mgL^− 1^	Chl a	Chl b	Car	Total pigments
0. 0	Control	0.0	1168.7 ± 2.06^e^	771.7 ± 1.63^e^	278.7 ± 1.76^b^	2219.2 ± 5.46^e^
	Esculin	50	1212.1 ± 2.01^d^	820.9 ± 1.86^d^	302.3 ± 3.17^b^	2335.3 ± 0.70^d^
		100	1287.6 ± 1.15^b^	962.1 ± 3.78^b^	327.3 ± 1.44^b^	2577.0 ± 4.04^b^
	Digitoxin	50	1258.0 ± 2.51^c^	882.7 ± 1.72^c^	314.8 ± 1.15^b^	2455.5 ± 5.39^c^
		100	1318.2 ± 1.47^a^	1014.7 ± 1.59^a^	323.3 ± 0.81^b^	2656.1 ± 2.88^a^
5000 mg/L	Control	0.0	827.2 ± 2.04^l^	555.8 ± 1.16^j^	297.7 ± 0.47^b^	1680.6 ± 3.68^ij^
	Esculin	50	883.3 ± 2.06^i^	615.3 ± 1.44^h^	316.2 ± 0.92^b^	1814.8 ± 4.43^h^
		100	950.3 ± 2.59^g^	651.6 ± 1.59^g^	332.1 ± 1.58^b^	1934.0 ± 2.59^g^
	Digitoxin	50	901.2 ± 3.14^h^	646.8 ± 0.57^g^	328.2 ± 3.65^b^	1876.2 ± 1.08^gh^
		100	980.2 ± 2.54^f^	684.4 ± 2.84^f^	343.6 ± 0.62^b^	2008.2 ± 4.76^f^

Results are means of three replicates. ± show SE

### Total soluble carbohydrates

**Fig. 1:**
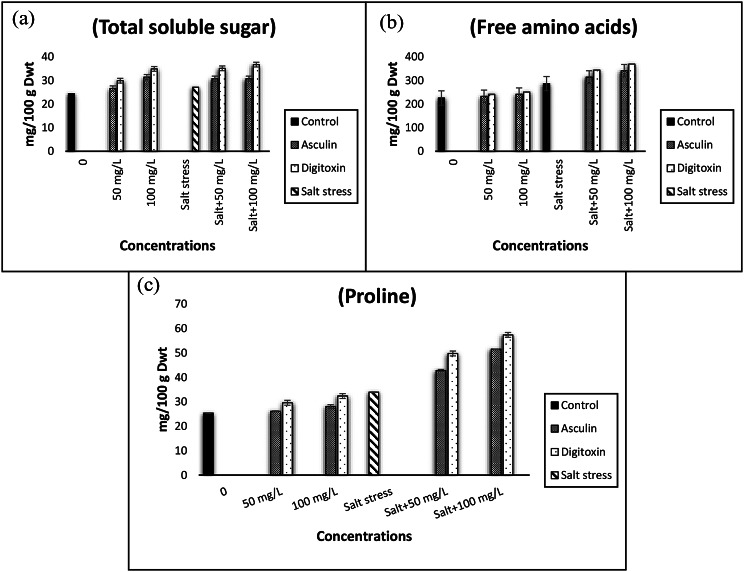
Impact of different concentrations of either esculin (**A**: 50, **B**: 100 mg/L) or digitoxin (**a**:50, **b**:100 mg/L) on total soluble sugar, free amino acids, and proline contents of flax plants growing under salt stress (0.0, 5000 mg/L). Results are means of three replicates. The bars on the columns show SE. The values with the same letter in the same column are non-significant and the values with different letters are significant

In the present study, a significant increase in total soluble carbohydrates was observed in salt-stressed flax plants (Fig. 1a). The application of either digitoxin or esculin significantly stimulated the accumulation of total soluble carbohydrates. The greatest accumulation was noticeable at high levels of digitoxin in salt-stressed-flax plants (Fig. 1a).

### Free amino acids and proline

Exposure of flax plants to salt stress induced an accumulation of free amino acids (Fig. 1b). Furthermore, digitoxin and esculin significantly enhanced the stimulatory role of salt stress on the production of free amino acids in flax plants. The accumulation of free amino acids is positively related to digitoxin and esculin concentrations. A maximum level of amino acids was displayed in salt-stressedplants treated with digitoxin. In addition, proline accumulation was observed in the green tops of all stressed flax plants compared with that of the control (unstressed) (Fig. 1c). In addition, the increases in proline levels were much higher in stressed plants (Fig. 1c). Notably, the proline content of the digitoxin and esculin-treated plants increased significantly, particularly under salt stress (Fig. 1c). Such increment in proline level was more pronounced in stressed plants treated with 100 mg/L digitoxin.

**Fig. 2:**
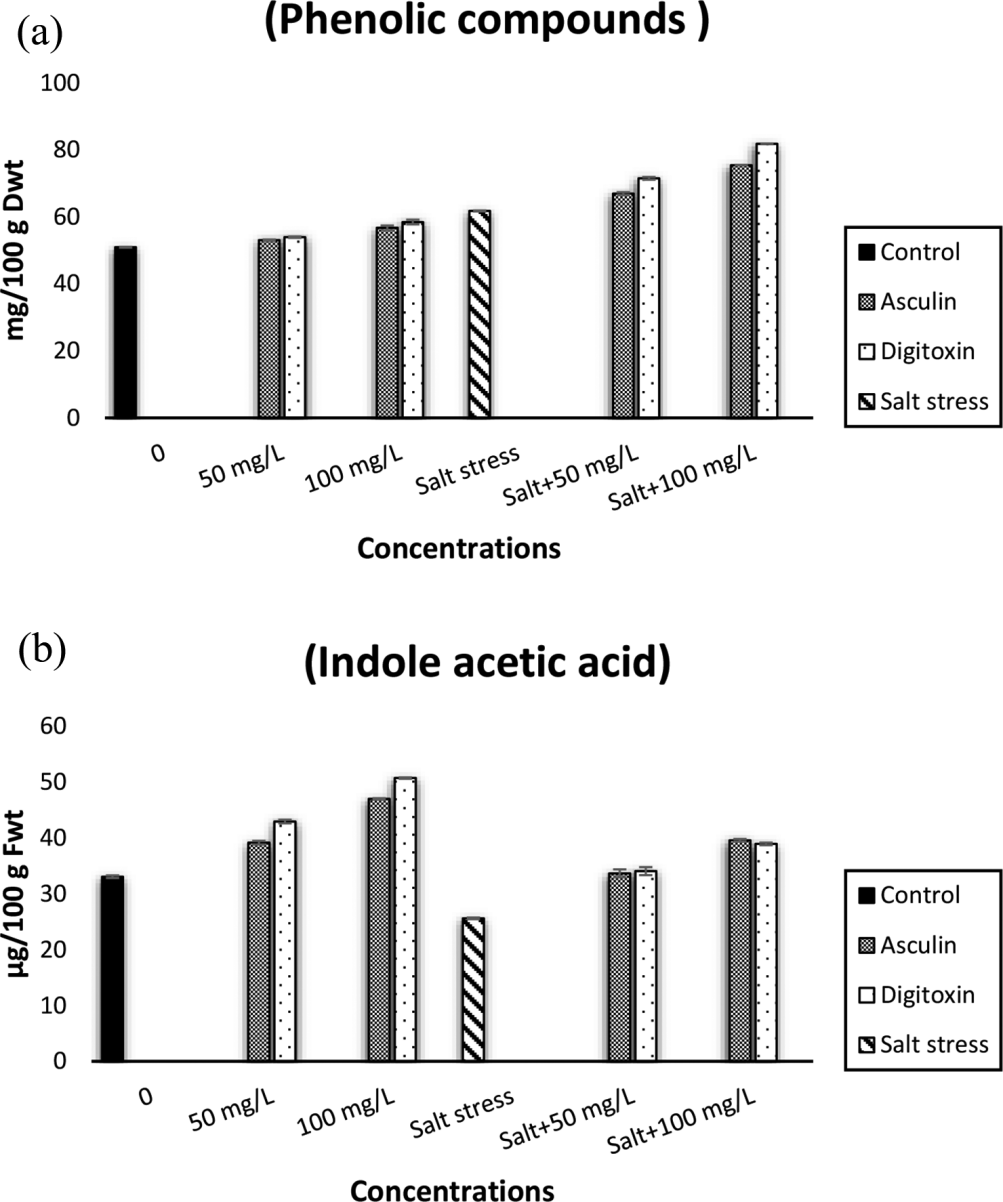
Impactof different concentrations of esculin (**A**: 50, **B**: 100 mg/L) or digitoxin (**a**: 50, **b**: 100 mg/L) on phenolic compounds andindole acetic acid contents of flax plants growing under control and salt stress (0.0, 5000 mg/L). Results are means of three replicates. The bars on the columns show SE. The values with the same letter in the same column are non-significant and the values with different letters are significant

### Total phenols

In the investigated study, the total phenolics of salt-stressed flax plants were significantly higher compared to that of control unstressed plants (Fig. 2a). Foliar application of flax plants with digitoxin or esculin markedly stimulated the accumulation of phenols, particularly under salt stress conditions. The accumulation of phenolic compounds was significantly higher in the plants treated with 100 mg/L of either digitoxin or esculin. The maximum level of phenol accumulation was estimated in salt stressed flax plants treated with a high dose of digitoxin.

### IAA level

The present results showed that the lowest IAA level was assayed in salt-stressed flax plants (Fig. 2b). However, digitoxin and esculin treatments significantly stimulated the accumulation of IAA levels in flax plants grown in unstressed and stressed environments. Such effects were positively related to the concentrations of digitoxin and esculin. The increase in IAA levels in salt-stressed flax plants after esculin or digitoxin treatments were significantly lower than the control plants treated with the same compounds. A greater amount of IAA was measured in digitoxin unstressed flax plants as compared to the rest of the treatments.

### Membrane stability index (MSI)

**Fig. 3:**
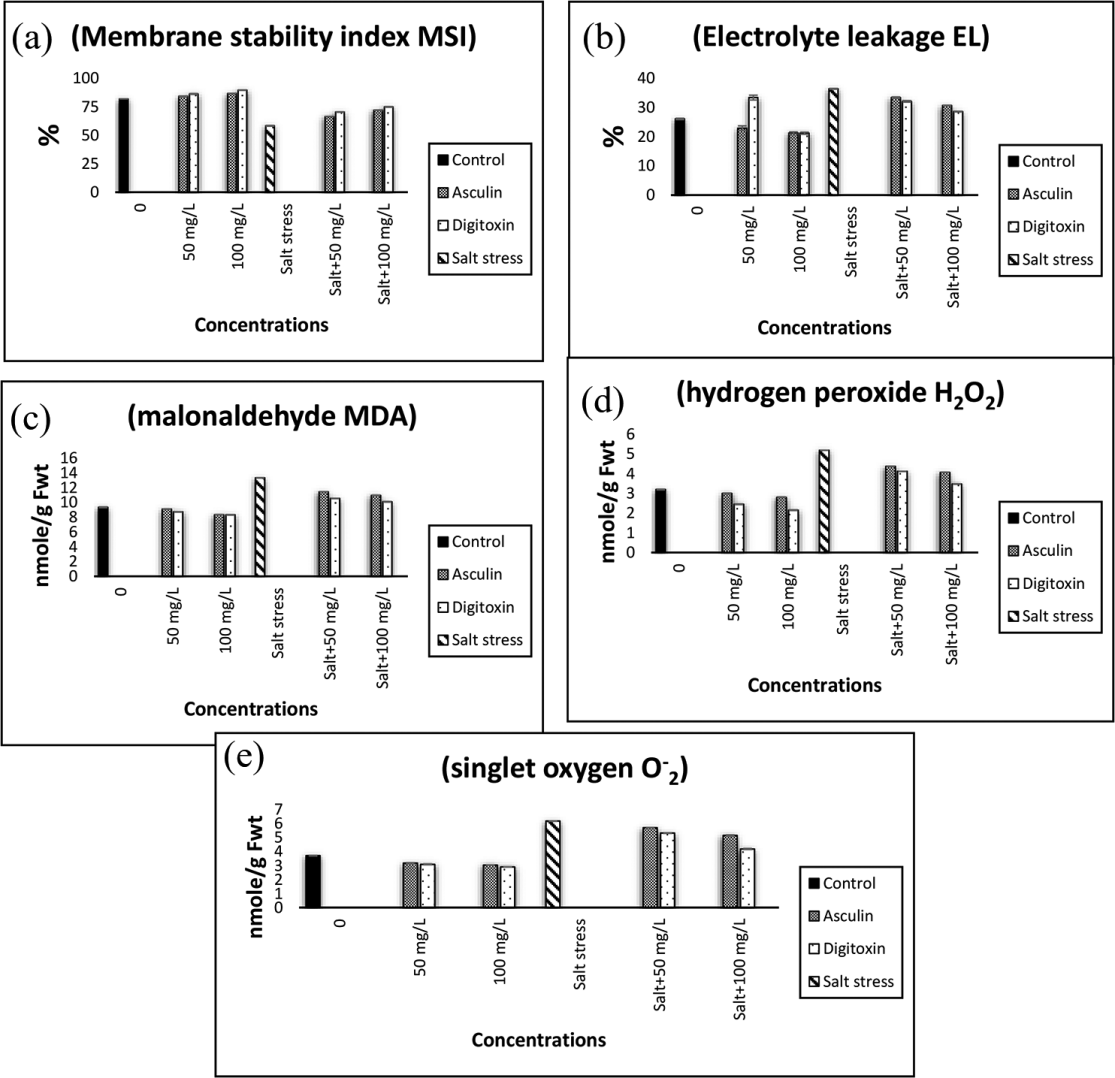
Impact of different concentrations of esculin (**A**: 50, **B**: 100 mg/L) or digitoxin (**a**: 50, **b**: 100 mg/L) on membrane stability index (MSI), electrolyte leakage (EL), malonaldehyde (MDA), hydrogen peroxide (H_2_O_2_), and singlet oxygen (O^−^ _2_) of flaxplants growing under salt stress (0.0, 5000 mg/L). Results are means of three replicates. The bars on the columns show SE. The values with the same letter in the same column are non-significant and the values with different letters are significant

The MSI of flax was markedly reduced after the imposition of salt stress (Fig. 3a). Exposure to either digitoxin or esculin significantly increased the MSI of flax plants. The increases in MSI values were greater in high dose treated plants grown under an unstressed normal environment. The maximum MSI was measured in stressed flax plants treated with 100 mg/L digitoxin as compared with the other conditions tested.

### Electrolyte leakage (EL)

The data presented in (Fig. 3b) showed the increments in EL in leaves of flax plants exposed to salinity stress. Exogenous application of either digitoxin or esculin at concentrations of 50 mg/L and 100 mg/L significantly reduced the EL values of stressed flax leaves compared to salt-stressed leaves. These reductions were more pronounced in high-dose digitoxin(at a concentration of 100 mg/L)treated leaves.

### Lipid peroxidation products

The extent of the salt-induced oxidative damage was assessed by measuring the levels of malondialdehyde (MDA) formation (Fig. 3c). Salinization significantly stimulated an increase in lipid peroxidation products in the shoots of flax plants.Exogenous application of either digitoxin or esculin at concentrations of 50 mg/L and 100 mg/L markedly reduced the peroxidation product (MDA). The values of peroxidation products are negatively related to the concentration of either digitoxin or esculin. Lower levels of MDA were estimated in digitoxin-treated plants, particularly at high doses.

### Reactive oxygen species

The results of this investigation showed that ROS including H_2_O_2_ and O_2_^•^levels are markedly increased in salt-stressed flax plants (Fig. 3d and e). Digitoxin and esculin glycoside treatments significantly reduced the accumulation of H_2_O_2_ and O_2_ in stressed and unstressed flax plants. These reductions were more visiblein plants exposed to the high dose of either digitoxin or esculin. Indeed, digitoxin at a concentration of 100 mg/L was the superior treatment that stimulated the reduction in the investigated ROS accumulation.

### Changes in antioxidants

#### Determination of antioxidant enzyme activities

Results in this study showed that SOD, catalase, and phenol peroxidases activities were markedly increased in salt – stressed fax plants (Table 5). The foliar application of either digitoxin or esculin significantly enhanced the antioxidant enzyme activities including SOD, catalase, and phenol peroxidases in salt-stressed flax plants. These stimulating effects were positively related to the concentrations of the investigated compounds. The maximum activities of the investigated antioxidant enzymes were assayed in high-dose digitoxin-treated plants exposed to salt stress. In contrast, higher lipoxygenase activity was assayed in salt-stressed flax plants (Table 5) and the treatments with either digitoxin or esculin significantly caused reductions in lipoxygenase activity, particularly under salt stress conditions. Such reduction was greater at high doses of either digitoxin or esculin.

#### Determination of tyrosine and phenylalanine ammonia-lyase activities

The activities of bothtyrosine ammonia-lyase (TAL) and phenylalanine ammonia- lyase (PAL) were significantly increased in salt-stressed flax shoot tops (Table 5). The treatment of flax plants with esculin or digitoxin significantly stimulated the activities of TAL and PAL, particularly under saline stress conditions (Table 5). Such increments in TAL and PAL activities were positively related to the esculin and digitoxin concentrations. The maximum activity of either TAL or PAL was recorded in stressed flax shoot tops exposed to 100 mg/L digitoxin.

**Table 5:** Impact of different concentrations of either esculin or digitoxin (50, 100 mg/L) on the activities of superoxide dismutase (SOD), catalase(CAT), peroxidase(POX), lipoxygenase, tyrosine ammonia lyase (TAL), and phenyl ammonia lyase (PAL) of flax plants in the absence or presence of salinity (0.0, 5000 g/L). Each value is the mean of three replicates ± SE with P value < 0.05.The values with the same letter in the same column are non-significant and the values with different letters are significant

Salinity mg/L	Treatment	Conc. mg/L	SOD	CAT	POX	Lipoxygenase	TAL	PAL
0.0	Control	0.0	41.23 ± 0.22^i^	37.05 ± 0.05^n^	13.63 ± 0.02^k^	8.81 ± 0.07^k^	4.51 ± 0.083^f^	1.54 ± 0.011^g^
	Esculin	50	43.93 ± 0.17^h^	41.88 ± 0.15^m^	15.79 ± 0.02^j^	8.19 ± 0.04^l^	5.75 ± 0.469^e^	2.40 ± 0.002^f^
		100	45.47 ± 0.11^gh^	45.34 ± 0.28^k^	17.71 ± 0.13^i^	7.31 ± 0.02^m^	6.61 ± 0.164^cd^	3.18 ± 0.004^d^
	Digitoxin	50	45.40 ± 0.14^gh^	43.40 ± 0.12^l^	17.71 ± 0.02^i^	7.48 ± 0.02^m^	6.20 ± 0.029^de^	2.30 ± 0.032^f^
		100	47.20 ± 0.02^g^	47.55 ± 0.23^j^	19.94 ± 0.17^h^	6.51 ± 0.01^n^	7.05 ± 0.056^c^	3.41 ± 0.143^c^
5000 mg/L	Control	0.0	46.83 ± 2.76^g^	48.76 ± 0.10^i^	19.90 ± 0.14^h^	15.69 ± 0.02^c^	6.19 ± 0.023^de^	2.63 ± 0.031^e^
	Esculin	50	53.83 ± 0.18^e^	53.93 ± 0.14^g^	28.46 ± 0.10^f^	13.33 ± 0.04^g^	7.11 ± 0.024^c^	3.46 ± 0.116^c^
		100	62.84 ± 0.17^b^	62.06 ± 0.45^c^	36.93 ± 0.14^b^	11.47 ± 0.02^i^	7.91 ± 0.035^b^	4.04 ± 0.048^b^
	Digitoxin	50	58.52 ± 0.06^d^	60.01 ± 0.47^e^	33.05 ± 0.05^d^	12.25 ± 0.05^h^	8.18 ± 0.018^b^	3.45 ± 0.052^c^
		100	66.63 ± 0.12^a^	64.34 ± 0.28^b^	40.88 ± 0.15^a^	10.64 ± 0.06^j^	9.07 ± 0.218^a^	4.28 ± 0.039^a^

Results are means of three replicates. ± show SE

### Mineral levels

Salt stress significantly reduced P, K, Ca, and Mg levels in salt-stressed flax shoots (Table 6). However, Na ions markedly increased in stressed plants (Table 6). Foliar application of either esculin or digitoxin at concentrations of 50 mg/L and 100 mg/ L significantly increased P, K, Ca, and Mg levels in the presence and absence of NaCl. These increases were concentration-dependent. The greatest increases in the previously mentioned elements were measured in digitoxin-treated plants. In contrast, the Na level was significantly decreased in esculin and digitoxin-treated plants, particularly at the high concentration of digitoxin.

**Table 6:** Impact of different concentrations of esculin or digitoxin (50, 100 mg/L) on potassium, sodium, phosphorous, calcium, and magnesium contents as well as the sodium/potassium ratio of flax plants in the absence and presence of salinity stress (0.0, 5000 mg/L). Each value is the mean of three replicates ± SE with P value < 0.05. The values with the same letter in the same column are non-significant and the values with different letters are significant

Salinitymg/L	Treatment	Conc mg/L	K	Na	Na/K	*P*	Ca	Mg
0.0	Control	0.0	26.20 ± 0.17^g^	5.68 ± 0.17^f^	0.22	13.56 ± 0.16^c^	9.23 ± 0.06^f^	6.12 ± 0.02^f^
	Esculin	50	32.26 ± 0.24^cd^	5.10 ± 0.02^i^	0.16	18.65 ± 0.17^ab^	12.83 ± 0.12^c^	7.73 ± 0.06^b^
		100	33.49 ± 0.36^b^	5.13 ± 0.12^i^	0.15	18.66 ± 0.008^ab^	13.41 ± 0.25^b^	7.66 ± 0.28^b^
	Digitoxin	50	33.06 ± 0.06^bc^	5.14 ± 0.002^hi^	0.16	18.52 ± 0.25^b^	12.91 ± 0.04^c^	7.36 ± 0.008^c^
		100	35.45 ± 0.28^a^	4.65 ± 0.17^j^	0.13	19.14 ± 0.11^a^	15.15 ± 0.11^a^	8.30 ± 0.02^a^
5000 mg/L	Control	0.0	20.96 ± 0.16^i^	7.24 ± 0.002^a^	0.35	7.31 ± 0.01^i^	6.23 ± 0.08^j^	5.09 ± 0.05^gh^
	Esculin	50	28.00 ± 0.54^f^	6.69 ± 0.08^b^	0.24	9.79 ± 0.25^f^	8.64 ± 0.17^g^	6.21 ± 0.07^f^
		100	30.01 ± 0.30^e^	6.30 ± 0.08^c^	0.21	11.37 ± 0.16^d^	9.73 ± 0.02^e^	6.48 ± 0.0001^e^
	Digitoxin	50	30.17 ± 0.40^e^	6.25 ± 0.05^cd^	0.21	11.75 ± 0.34^d^	9.20 ± 0.08^f^	6.66 ± 0.10^e^
		100	31.81 ± 0.38^d^	5.41 ± 0.04^g^	0.17	13.95 ± 0.11^c^	10.86 ± 0.12^d^	6.95 ± 0.09^d^

Results are means of three replicates. ± Show SE

#### Yield components

Data presented in Table 7 showed that digitoxin and esculin treatments markedly stimulated shoot growth during the mature stage as evidenced by the increments in shoot length, technical stem length (cm), and shoot dry weight in stressed plants as compared with untreated stressed flax plants. Such increments in the investigated growth parameters were concentration dependent. The highest values of shoot height, technical length(The length of shoot system till the fruiting branches), and weight were measured in digitoxin-treated shoots compared with the other treatments. Moreover, yield components such as fruiting zone length (cm), the number of fruiting branches/plant, the number of fruits/plant, seed yield/plant (g), seed yield (kg per plant), 1000 seeds weight (g), were significantly increased in esculin and digitoxin treated plants (Table 7) under stress and normal conditions. The increments in the previously mentioned parameters were more pronounced in digitoxin-treated plants, particularly at high concentrations (Table 7).

**Table 7:** Impact of different concentrations of esculin or digitoxin (50, 100 mg/L) on yield and its components of flax plants in the absence and presence of salinity stress (0.0, 5000 mg/L). Each value is the mean of three replicates ± SE with P value < 0.05. The values with the same letter in the same column are non-significant and the values with different letters are significant

Salinity	Treatments	Conc mg/L	Shoot length (cm)	Fruit zoneLength (cm)	Technical Shootlength cm	Number of Fruiting branches/plant	Number of fruits/plant
S 0	Control	0	55.33 ± 0.33^ef^	10.66 ± 0.66^ef^	44.66 ± 0.88^ef^	6.33 ± 0.33^f^	11.00 ± 0.57^ef^
	Esculin	50	62.66 ± 1.20^d^	14.66 ± 0.88^ab^	48.00 ± 0.57^de^	7.00 ± 0.57^ef^	12.67 ± 0.33^cd^
		100	72.00 ± 0.57^b^	15.66 ± 0.33^a^	56.33 ± 0.66^b^	9.67 ± 0.33^b^	14.33 ± 0.66^b^
	Digitoxin	50	67.66 ± 0.66^c^	13.33 ± 0.33^bc^	54.33 ± 0.88^bc^	8.67 ± 0.33^bcd^	13.66 ± 0.33^bc^
		100	83.66 ± 1.33^a^	15.66 ± 0.66^a^	68.00 ± 1.15^a^	11.66 ± 0.33^a^	16.67 ± 0.33^a^
S1 5000	Control	0	41.66 ± 0.66^i^	8.66 ± 0.33^g^	33.00 ± 1.00^j^	4.67 ± 0.33^g^	7.33 ± 0.33i
	Esculin	50	48.33 ± 0.66^h^	10.67 ± 0.33^ef^	37.66 ± 0.88^i^	6.33 ± 0.33^f^	10.33 ± 0.66^efg^
		100	57.66 ± 0.88^e^	12.66 ± 0.33^cd^	45.00 ± 0.57^ef^	8.00 ± 0.57^cde^	11.33 ± 0.33^de^
	Digitoxin	50	53.00 ± 1.15^fg^	12.67 ± 0.33^cd^	40.33 ± 1.20^ghi^	7.33 ± 0.33^def^	11.33 ± 0.33^de^
		100	65.00 ± 1.15^cd^	13.66 ± 0.33^bc^	51.33 ± 1.45^cd^	9.00 ± 0.001^bc^	13.66 ± 0.33^bc^
**Salinity**	**Materials**	**Conc mg/L**	**Shoot dry wt g**	**Pods wt/ plant (g)**	**Seeds wt /plant (g)**	**1000 seeds wt (g)**	
S 0	Control	0.0	3.03 ± 0.02^e^	0.69 ± 0.022^d^	0.46 ± 0.002^c^	5.25 ± 0.03^d^	
	Esculin	50	4.02 ± 0.12^c^	0.83 ± 0.014^bc^	0.56 ± 0.02^b^	5.88 ± 0.07^c^	
		100	4.69 ± 0.07^b^	0.85 ± 0.005^b^	0.65 ± 0.005^a^	6.20 ± 0.03^b^	
	Digitoxin	50	4.15 ± 0.03^c^	0.81 ± 0.005^c^	0.58 ± 0.003^b^	6.18 ± 0.03^b^	
		100	5.08 ± 0.10^a^	0.94 ± 0.012^a^	0.64 ± 0.039^a^	7.18 ± 0.01^a^	
S 5000	Control	0.0	1.98 ± 0.07^i^	0.33 ± 0.003^i^	0.31 ± 0.007^fg^	3.32 ± 0.06^i^	
	Esculin	50	2.58 ± 0.12^g^	0.43 ± 0.006^g^	0.37 ± 0.003^de^	3.88 ± 0.03^h^	
		100	3.10 ± 0.03^e^	0.51 ± 0.005^f^	0.39 ± 0.007^de^	4.41 ± 0.03^f^	
	Digitoxin	50	2.58 ± 0.02^g^	0.50 ± 0.002^f^	0.37 ± 0.008^e^	4.11 ± 0.008^g^	
		100	3.44 ± 0.05^d^	0.62 ± 0.002^e^	0.41 ± 0.008^d^	5.09 ± 0.02^e^	

Results are means of three replicates. ± show SE

#### Chemical composition of seeds

Table 8 showed the seed quality of untreated and treated with either sculin or digitoxin-flax plants in terms of oil (%), carbohydrates (%), and protein content. The exposure of flax plants to 50 mg/L or 100 mg/L of either esculin or digitoxin significantly improved the quality of flax seeds as estimated by oil (%), carbohydrates (%), and protein content. Such increments in the previous parameters were pronounced in unstressed and stressed plants treated with 100mg/L digitoxin as compared with their reference controls (normal grown and NaCl stressed plants) as well as esculin treatments.

**Table 8:** Impact of different concentrations of esculin or digitoxin (50, 100 mg/L) on oil, carbohydrate (CHO), and protein contents of flax seeds in the absence and presence of salinity stress (0.0, 5000 mg/L). Each value is the mean of three replicates ± SE with P value <0.05. The values with the same letter in the same column are non-significant and the values with different letters are significant

Salinity treatment	glycoside	Conc. mg/L	Oil%	CHO%	Protein%
0.0	Control	0	36.4 ± 0.14^d^	32.6 ± 0.15^e^	16.3 ± 0.03^f^
	Esculin	50	36.7 ± 0.04^c^	33.1 ± 0.02^d^	17.1 ± 0.05^c^
		100	37.1 ± 0.02^b^	34.2 ± 0.02^b^	17.3 ± 0.08^b^
	Digitoxin	50	37.0 ± 0.07^b^	33.7 ± 0.06^c^	17.1 ± 0.05^c^
		100	37.3 ± 0.02^a^	35.0 ± 0.07^a^	17.5 ± 0.04^a^
5000 mg/L	Control	0	33.7 ± 0.06^i^	30.3 ± 0.08^h^	15.2 ± 0.02^j^
	Esculin	50	34.1 ± 0.02^h^	31.7 ± 0.03^f^	15.9 ± 0.02^g^
		100	34.7 ± 0.04^f^	31.8 ± 0.21^f^	16.2 ± 0.03^f^
	Digitoxin	50	34.4 ± 0.10^g^	33.1 ± 0.03^d^	16.0 ± 0.005^g^
		100	35.6 ± 0.05^e^	33.8 ± 0.08^c^	16.3 ± 0.02^f^

#### Fatty acid profile of the yielded oil

Gas-liquid chromatography analysis revealed the presence of Lauric (C12:0), Myristic (C 14:0) palmitic (C16:0), Stearic (C18:0), Oleic (C18:1), Linoleic (C18:2), Linolenic (C18:3), Behenic (C 22:0) and Lignoceric (C 24:0) fatty acids in flax seed oil (Table 9). Meanwhile, the major saturated fatty acids in flax seed oil were palmitic acid and stearic acid. However, Linolenic and Oleic acids were the most abundant unsaturated fatty acids. The present results revealed that imposition of salinity causes increases in saturated fatty acids including palmitic (C16:0), Stearic (C18:0), Behenic (C 22:0), and Lignoceric. On the other hand, reductions in unsaturated fatty acids were detected in salt-stressed oil seeds compared with control plants. In addition, the exposure of flax plants to either esculin or digitoxin markedly increases the levels of unsaturated fatty acids particularly Oleic and Linoleic acids (Table 9). On the other hand, saturated fatty acids markedly decreased.

**Table 9:** Impact of 100 mg/L of esculin or digitoxin on the fatty acids profile of salt-stressed flax seeds (0.0, 5000 mg/L). The values with same letter in the same column are non-significant and the values with different letters are significant

Fatty acid (%)	Control	Salt (5000 mg/L)	Esculin(100 mg/L) + Salt (5000 mg/L)	Digitoxin (100 mg/L) + Salt (5000 mg/L)
Lauric C12:0	0.25	1.45	1.02	1.07
Myristic C14:0	1.35	2.34	2.00	2.12
Palmitic C16:0	9.35	10.07	9.62	9.58
Stearic C18:0	4.08	4.68	4.25	4.12
Oleic C18:1	16.80	15.98	16.35	16.80
Linoleic C18:2	19.19	18.24	19.00	18.60
Linolenic C18:3	45.10	42.64	44.16	45.30
Behenic C22:0	1.69	2.14	1.35	0.19
Lignoceric C24:0	2.19	2.46	2.25	2.22
TSFA	18.91	23.14	20.49	19.30
TUSFA	81.09	76.86	79.5 1	80.70
TUS/TS	4.29	3.32	3.88	4.18

Results are means of three replicates. ± show SE

### Straw yield

It was also noticed that the straw yield significantly increased in response to esculin and digitoxin treatments (Table 10). The maximum increase in straw yield per plant was recorded in plants exposed to 100 mg/L digitoxin under unstressed and stressed conditions as compared with their corresponding controls. Notably, the present results also showed that fiber length, width, and fiber percentage were markedly increased in the straw of esculin, and digitoxin-treated plants exposed to normal and salt condition (Table 10). The greatest increases were measured at high concentrations of either esculin or digitoxin. Meanwhile, the high concentration of digitoxin improved the fiber length, width, and fiber % as compared with the other treatments.

**Table 10:** Impact of different concentrations of esculin or digitoxin (50, 100 mg/L) on fiber length, fiber weight, straw yield, and fiber percentage contents of flax plants in the absence and presence of salinity stress (0.0, 5000 mg/L). Each value is the mean of three replicates ± SE with P value < 0.05. The values with same letter in the same column are non-significant and the values with different letters are significant

Salinity treatment	Glycoside	Conc mg/L.	Fiber length (cm)	Fiber wt/plant (g)	Straw yield/plant (g)	Fiber %
0.0	Control	0.0	53.66 ± 0.33^e^	0.25 ± 0.008^f^	1.47 ± 0.04^g^	17.22 ± 1.11^cd^
	Esculin	50	55.00 ± 2.08^e^	0.35 ± 0.006^cd^	1.78 ± 0.08^f^	20.10 ± 1.07^a^
		100	67.67 ± 0.33^a^	0.43 ± 0.003^b^	2.35 ± 0.05^cd^	18.57 ± 0.31^abcd^
	Digitoxin	50	55.00 ± 0.57^e^	0.42 ± 0.013^b^	2.13 ± 0.02^e^	19.84 ± 0.57^a^
		100	60.66 ± 0.66^cd^	0.49 ± 0.008^a^	2.53 ± 0.04^ab^	19.48 ± 0.38^ab^
5000 mg/L	Control	0.0	55.00 ± 1.00^e^	0.31 ± 0.005^e^	1.82 ± 0.07^f^	17.01 ± 0.56^d^
	Esculin	50	59.67 ± 0.88^d^	0.38 ± 0.014^c^	2.13 ± 0.03^e^	17.96 ± 0.54^abcd^
		100	62.67 ± 0.33^bc^	0.48 ± 0.003^a^	2.43 ± 0.04^bc^	19.85 ± 0.51^a^
	Digitoxin	50	64.00 ± 0.57^b^	0.42 ± 0.017^b^	2.24 ± 0.02^de^	19.01 ± 0.97^abcd^
		100	55.00 ± 1.00^e^	0.48 ± 0.003^a^	2.61 ± 0.01^a^	18.49 ± 0.08^abcd^

Results are means of three replicates. ± show SE

**Fig. 4:**
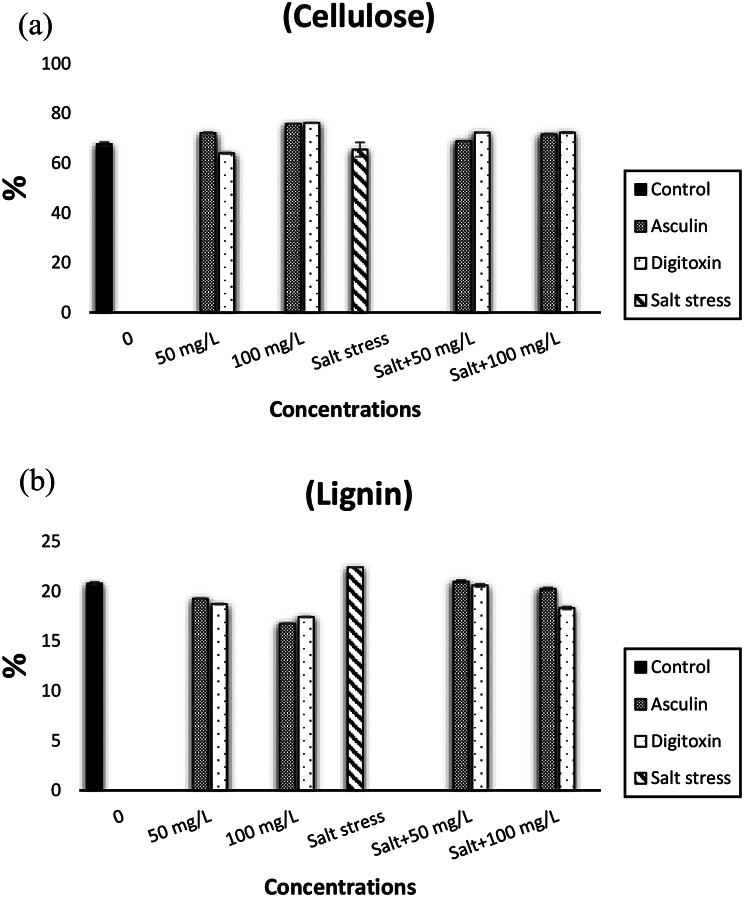
Impact ofdifferent concentrations of esculin (A: 50, B: 100 mg/L) or digitoxin (a: 50, b: 100 mg/L) on cellulose (**a**) and lignin (**b**) contents of flax plants growing under salt stress (0.0, 5000 mg/L). Results are means of three replicates. The bars on the columns show SE. The values with the same letter in the same column are non-significant and the values with different letters are significant

Indeed, the changes in fiber constituents in terms of cellulose and lignin were illustrated in Fig. 4a and b. The celluloselevel was markedly reduced in salt-stressed straws compared with control plants. On the other hand different concentrations of either esculin and digitoxin markedly increased cellulose levels either under normal and stressed conditions (Fig. 4a). On the contrary, the imposition of salt stress significantly enhanced the lignin percentage as compared with the normal unstressed condition (Fig. 4b). Application of either esculin or digitoxin at 50 and 100 mg/L significantly reduced the percentage of lignin in flax straw grown in a normal and stressed environment.

## Discussion

Salinity stress is an extremely complicated problem that interrupts plant growth, development, and thereby productivity [55 & 56]. Esculin and Digitoxin are two compounds consisting of a coumarin moiety that is glycosidically linked to a carbohydrate molecule. As a result, esculin demonstrated strong antioxidant capabilities, preventing triglycerides from auto-oxidizing [72], and preserving the integrity of the cell membrane. Furthermore, digitoxin decreased Na uptake and elevated K, Ca, P, and Mg in flax plants by using cardiac glycosides as selective inhibitors of Na^+^/K + ATPase (EC 3.6.3.9).According to a theory, these substances help to create and preserve the electrochemical gradient across the plasma membrane, which is essential for many inorganic and organic substrates’ secondary transport as well as physiological functions like osmotic regulation and ion homeostasis [23].This enhances the growth and productivity of flax plants. According to a report, the presence of sugars in these compounds enhances the activity of cardiac glycosides several times, and the structure of the sugar or sugars has a significant impact on the activity of the cardiac glycoside [76].In the present investigation, the exposure of flax plants to salt stress significantly impaired the morphological parameters(Table 3). These results were in accordance with those obtained by Abdullah [54]. Moreover, salinity reduced stomatal conductance and disrupted photosystems, thus promoting the accumulation of ROS such as superoxide and hydrogen peroxide, which stimulated the oxidative degradation of chlorophyll a and b (Table 4) and enhanced membrane peroxidation as evidenced by the significant accumulation of malonaldehyde and caused a greater increase in electrolyte leakage (Fig. 3b and c). Meanwhile, the membrane stability index significantly decreased in stressed flax shoots (Fig. 3a).MSI of flax leaves is a widely used criterion to assess salt tolerance, since salinity causes water loss from plant tissues, which seriously impairs both membrane structure and function. Similar results have been obtained by El-Bassiouny and Sadak [56], , Alaei [52], and Abdullah [55] on flax plants.Salt stress-induced ROS overproduction is one of the major reasons for devastating the morphological, physiological, and biochemical activities of plants, as well as reducing stomatal conductance and disrupting photosystems [57]. In addition, the decline in chlorophyll contents in salt-stressed flax leaves could be attributed to the destruction of chlorophylls via the chlorophyllase enzyme or/and the instability of pigment-protein complexes [5, 58]. It was reported that the overproduction of reactive oxygen species disturbs the homeostasis of cells, causing lipid peroxidation and destruction of the membranes, thereby increasing membrane leakage, affecting cell viability [59], and, consequently, reducing crop productivity [6]. Meanwhile, the foliar application of either esculin (antioxidant coumarin glycosides) or digitoxin (a Na/K inhibitor)significantly nullified the salt stress hazards and restored the growth of flax plants via buffering ROS, thereby maintaining membrane integrity, as evidenced by reductions of both MDA and EL. The reduction in H_2_O_2_ and O^•2−^ levels in flax plants was concomitant with increments in pigment levels and thereby all investigated morphological parameters, particularly in high-dose digitoxin-treated flax plants (Fig. 3d and e).

Notably, plants have developed various defense mechanisms to cope with the adverse effects of salt stress. The antioxidant defense system is one of the most important mechanisms involved in the mitigation of salinity-induced injuries displayed by ROS [1]. The present results showed that salt-stressed flax shoots exhibited high levels of carotenoids (Table 4). In addition, salinity stimulated the activities of SOD, CAT, and peroxidase as well as the lipoxygenase enzyme in flax shoots (Table 5). Similar results have been reached by Ahmad [60] and Sadak [61] on different plant species. Phenylalanine is involved in the defense response of plants, and it is commonly used as a stress indicator. PAL is the key regulatory enzyme in the phenylpropanoids pathway [62]. PAL is involved in “switching” from the primary to the secondary metabolism of the plant and leads to the formation of a wide range of secondary metabolites [63]. The imposition of salt stress resulted in the stimulation of PAL and TAL activities in flax plants (Table 5). Foliar application of either esculin or digitoxin significantly stimulated the antioxidant enzyme activities, including SOD, POX, and catalase as well as PAL and TAL (Table 5) enzymes, under salt stress conditions. Recently, it was reported that PAL and TAL contribute to the biosynthesis and accumulation of secondary metabolites such as phenols and lignin [64]. The effect of salinity on PAL and TAL on salt-stress tobacco was also investigated by Mohagheghian and Ehsan Pour [65]. On the contrary, the lipoxygenase activity was markedly decreased in the glycoside-treated flax plants exposed to salt stress as compared with their reference controls (Table 5). The reduction in lipoxygenase activity was positively related to MDA levels as well as EL.

In addition, the accumulation of compatible solutes in treated stressed flax plants(Fig. 1), sustains the ionic balance of the vacuole, neutralizes ROS, and thereby protects macromolecules and plant organelles from the severe effects of oxidative stress [11, 53]. It was reported that proline has accumulated in many stressed plant species [8 & 12]. Moreover, esculin or digitoxin-treated flax plants exhibited greater levels of compatible solutes, including carbohydrates, free amino acids, and proline, particularly in stressed plants exposed to the high dose of digitoxin (Fig. 1). Notably, proline can serve as a compatible solute, osmo-protectant, source of nitrogen and carbon, membrane stabilizer, and ROS buffering scavenger [11& 68]. It was also stated that the accumulated soluble sugars utilized as osmolytes, as well as antioxidant compounds, thereby contribute to the enhancement of cell membrane stability [66].

Furthermore, salinity stress stimulated the accumulation of phenols in flax shoots (Fig. 2a). Such increments in phenols were more pronounced in both esculin and digitoxin-treated flax plants exposed to salt stress. The increment in the accumulation of free phenols in salt-stressed plants might be due to the greater stimulation of phenols biosynthesis [67]. It was reported that phenols play a crucial role in plant–protection against environmental stress due to their antioxidant activity [68]. Furthermore, ion homeostasis particularly K^+^/ Na^+^ is necessary for improving salt-stressed tolerance. It was reported that the uptake, efflux, translocation, and compartmentation of toxic ions particularly, Na^+^ offer the most important issue for salinity tolerance in plants, and consequently crop productivity [69]. Salinity-induced ionic imbalance in flax shoots is evidenced by the accumulation of greater levels of Na which impaired the accumulation of K, Ca, Mg, and P ions as compared with those of control unstressed flax plants (Table 6). Salinity significantly altered the cation mineral profiles of flax plants (Table 6). The imbalance of Na^+^ and other cations resulted in undesired ratios of Na^+^/K^+^ and Na^+^/Ca^2+^. Notably, the ionomic profile of flax is affected by element availability, uptake, transport, and environmental stress [70]. The accumulation of excess Na^+^ competitively inhibits the uptake of some other cations, including K^+^, Ca^2+^, and Mg^+^, P thus leading to an imbalance in cellular homeostasis, oxidative stress, and interference with Ca^2+^ and K^+^ functions [71]. Data of the present investigation showed that foliar application of digitoxin increased nutrient levels in salt-stressed flax shoots, particularly K, Ca, and P, however, decreased Na levels thereby inducing ion and nutrient balance and consequently mitigating salt stress hazards in flax plants. The protective roles of esculin were due to its good antioxidant properties which involved protecting triglycerides against auto-oxidation [73], however, digitoxin might be able to maintain ion homeostasis via inhibition of Na uptake and increased K uptake and thereby improvethe ability to maintain stable plasma membrane (PM) potentials [20].

Notably, Na^+^ interferes with K^+^ homeostasis, so maintaining a balanced cytosolic Na^+^/K^+^ ratio has been used as a crucial salinity tolerance strategy. In particular, the imbalance in Na^+^/K^+^ and Na^+^/Ca^2+^ ratios altered the plant’s physiological traits, including plant growth and photosynthesis [73]. It has been indicated that plant tolerance under salt stress requires a high cytosolic K^+^ /Na^+^ ratio in the cytoplasm. Similar results have been reached by Kim et al. [71].A higher Na^+^/ K^+^ ratio is an indicator of salt sensitivity, and this suggests that Na+-mediated damage to plants [71].

In addition, salt reduced endogenous IAA levels in flax shoots (Fig. 2b). This reduction might be due to salt-induced IAA degradation and /or reduction in its biosynthesis [74]. Such results are in accordance with those of Sadak [75]. In the contrast, foliar application of either esculin or digitoxin markedly increased the endogenous levels of IAA in stressed and unstressed flax shoots. The increments in IAA levels in unstressed and stressed flax plants treated with esculin and digitoxin might be attributed to glycoside moiety which might be involved in the improvement of biosynthesis of IAA and/or their antioxidant activity which protected IAA from oxidation.

Yield and yield components of flax plants treated with foliar application of cardiac glycosides digitoxin and antioxidant glycoside esculin are shown in Tables 1 and 2. Digitoxin and esculin treatments markedly stimulated different yield parameters under stress and normal conditions. Such enhanced effects might be due to presence of sugar moiety in these compounds. It was reported that the activity of cardiac glycosides is enhanced several-fold due to the presence of the sugars in these compounds as well as the structure of the sugar [76].

Furthermore, esculin and digitoxin significantly improved the quality of flax seeds as estimated by oil%, carbohydrates%, and proteins% (Table 2). The stimulatory effect of esculin and digitoxin might be attributed to the increase in endogenous hormonescontent (particularly IAA) which induces linear growth and development of plants [75]. Moreover, esculin and digitoxin exhibited antioxidant activity which stimulates the biosynthesis of photosynthetic pigments and thereby metabolites synthesis content which could lead to an increase in seeds weight. In addition, the increases in oil% could be attributed to the increase in the growth in vegetative parameters and nutrient uptake [77].

Gas-liquid chromatography analysis revealed that salinity stress caused increases in saturated fatty acids including palmitic, Stearic, Behenic, and Lignoceric. Meanwhile, reduced unsaturated fatty acids compared with control plants. These obtained results have been reached also by many investigators who stated that the unsaturated fatty acids content decreases with stress in some oil crops [53, 78 & 79]. It was reported that salinity stress at the seed development stage was found to decrease photosynthetic assimilation and carbon partitioning to seeds and increase the activities of enzymes involved in fatty acid oxidation [80]. In addition, the exposure of flax plants to either esculin or digitoxin markedly increases the levels of unsaturated fatty acids particularly Oleic and Linoleic acids (Table 10). On the other hand, saturated fatty acids markedly decreased. Ramadan [81] reported that the percentage of unsaturated fatty acids proved the efficiency of desaturation in oil. Indeed, the increments in the total unsaturated/saturated fatty acids ratio (TUS/TS) by esculin and digitoxin in the yielded oil become more favorable for human consumption. Polyunsaturated fatty acid (PUFA) lowers the risk of diseases related to cholesterol oxidation.

It was also noticed that the straw yield significantly increased in response to esculin and digitoxin treatments (Table 9). Notably, the present results also showed that fiber length, width, and fiber percentage were markedly increased in the straw of esculin, and digitoxin-treated plants exposed to normal and salt condition (Table 9). Indeed, the changes in fiber constituents in terms of cellulose and lignin were illustrated in Fig. (4a & 4b). On contrary, the application of either esculin or digitoxin significantly reduced the percentage of lignin in flax straw grown in normal and stressed environments (Fig. 4b). The reductions in lignin content of treated straw were positively related to the glycoside concentrations. Meanwhile, cellulose percentage was markedly increased in esculin and digitoxin-treated straw at the two investigated concentrations (Fig. 4a). In salt-stressed treated plants, a decrease in several physiological and metabolic processes, such as water uptake, chlorophyll content, photosynthesis, transpiration rate, nutrient availability, stomata conductance, and root hydraulic conductance, coincided with a reduction in growth and yielded seed percentage as well as straw yield [27 & 83].

## Conclusions

Data of the present investigation revealed that foliar application of either esculin or digitoxin at concentrations 50 mg L^− 1^ and 100mg L^− 1^ significantly enhanced the uptake of essential nutrients (K, Mg & P) and activated the antioxidant defense system which in turn detoxified ROS as well as stimulated the accumulation of osmoprotectantsthereby protected cell membranes and organelles particularly chloroplasts from salt adverse effects. Allthese investigated biochemical strategies were closely related to the observed increments in flax growth and yield components as well as improving the oil and straw quality and quantity under salt stress conditions. The present work suggested that the application of Na inhibitor digitoxin was more effective in enhancing stress tolerance, particularly at the high concentration compared to the antioxidant esculin glycosides. In conclusion, this investigation suggested that inhibition of Na accumulation by digitoxin accompanied by its antioxidant properties was more efficient in improving flax salt tolerance compared to ROS buffering antioxidant esculin.

## Data Availability

The authors declare that all data generated or analyzed during this study are included in this published article.
